# Transatlantic analysis of patient profiles and mid-term survival after isolated coronary artery bypass grafting: a head-to-head comparison between the European DuraGraft Registry and the US STS Registry

**DOI:** 10.3389/fcvm.2024.1366460

**Published:** 2024-09-12

**Authors:** Etem Caliskan, Martin Misfeld, Sigrid Sandner, Andreas Böning, Jose Aramendi, Sacha P. Salzberg, Yeong-Hoon Choi, Louis P. Perrault, Ilker Tekin, Gregorio P. Cuerpo, Jose Lopez-Menendez, Luca P. Weltert, Johannes Böhm, Markus Krane, José M. González-Santos, Juan-Carlos Tellez, Tomas Holubec, Enrico Ferrari, Gheorghe Doros, Maximilian Y. Emmert

**Affiliations:** ^1^ Department of Cardiothoracic and Vascular Surgery, Deutsches Herzzentrum der Charité (DHZC), Berlin, Germany; ^2^ Charité-Universitätsmedizin Berlin, Corporate Member of Freie Universität Berlin and Humboldt-Universität zu Berlin, Berlin, Germany; ^3^ University Department of Cardiac Surgery, Leipzig Heart Center, Leipzig, Germany; ^4^ Royal Prince Alfred Hospital, Sydney, Australia; ^5^ Institute of Academic Surgery at RPA, Sydney, Australia; ^6^ The Baird Institute of Applied Heart and Lung Surgical Research, Sydney, Australia; ^7^ Medical School, University of Sydney, Sydney, NSW, Australia; ^8^ Department of Cardiac Surgery, Medical University of Vienna, Vienna, Austria; ^9^ Department of Cardiovascular Surgery, Justus Liebig University Giessen, Giessen, Germany; ^10^ Division of Cardiac Surgery, Hospital de Cruces, Barakaldo, Spain; ^11^ Swiss Ablation, Herz & Rhythmus Zentrum AG, Zurich, Switzerland; ^12^ Kerckhoff Heart Center Bad Nauheim, Campus Kerckhoff Justus-Liebig University, Giessen, Germany; ^13^ Department of Cardiac Surgery, Montreal Heart Institute, Montreal, Canada; ^14^ Department of Cardiovascular Surgery, Manavgat Government Hospital, Manavgat, Turkey; ^15^ Department of Cardiovascular Surgery, Faculty of Medicine, Bahçeşehir University, Istanbul, Turkey; ^16^ Department of Cardiac Surgery, Hospital General Universitario Gregorio Marañón, Madrid, Spain; ^17^ Department of Cardiac Surgery, Hospital Universitario Ramon y Cajal, Madrid, Spain; ^18^ Department of Cardiac Surgery, European Hospital, Rome, Italy; ^19^ Department of Cardiovascular Surgery, German Heart Center Munich, Munich, Germany; ^20^ Division of Cardiac Surgery, Department of Surgery, Yale School of Medicine, New Haven, CT, United States, United States; ^21^ Department of Cardiovascular Surgery, Hospital Universitario de Salamanca, Salamanca, Spain; ^22^ Department of Cardiovascular Surgery, Hospital Universitario Virgen Macarena, Seville, Spain; ^23^ Department of Cardiovascular Surgery, University Hospital and Gothe University Frankfurt, Frankfurt/Main, Germany; ^24^ Department of Cardiovascular Surgery, Cardiocentro Ticino Institute, EOC, Lugano, Switzerland; ^25^ Department of Biostatistics, Boston University, School of Public Health, Boston, MA, United States; ^26^ Boston Clinical Research Institute (BCRI), Boston, MA, United States

**Keywords:** CABG, outcome, Europe, United States, mortality

## Abstract

**Introduction:**

Although cardiovascular surgery societies in Europe and the USA constantly strive for the exchange of knowledge and best practices in coronary artery bypass grafting (CABG), the available evidence on whether such efforts result in similar patient outcomes is limited. Therefore, in the present analysis, we sought to compare patient profiles and overall survival outcomes for up to 3 years between large European and US patient cohorts who underwent isolated CABG.

**Methods:**

Patients from the European DuraGraft Registry (*n* = 2,522) who underwent isolated CABG at 45 sites in eight different European countries between 2016 and 2019 were compared to randomly selected patients from the US STS database who were operated during the same period (*n* = 294,725). Free conduits (venous and arterial grafts) from the DuraGraft Registry patients were intraoperatively stored in DuraGraft, an endothelial damage inhibitor, before anastomosis, whereas grafts from the STS Registry patients in standard-of-care solutions (e.g., saline). Propensity score matching (PSM) models were used to account for differences in patient baseline and surgical characteristics, using a primary PSM with 35 variables (2,400 patients matched) and a secondary PSM with 25 variables (2,522 patients matched, sensitivity analysis). The overall survival for up to 3 years after CABG was assessed as the primary endpoint.

**Results:**

The comparison of patient profiles showed significant differences between the European and US cohorts. The European patients had more left main disease, underwent more off-pump CABG, and received more arterial grafts together with more complete arterial grafting procedures. In contrast, the US patients received more distal anastomoses with more saphenous vein grafts (SVGs) that were mainly harvested endoscopically. Such differences, however, were well balanced after PSM for the mortality comparison. Mortality comparison at 30 days, 12 months, and 24 months between the European and US patients was 2.38% vs. 1.96%, 4.32% vs. 4.79%, and 5.38% vs. 6.96%, respectively. At 36 months, the mortality was significantly lower in the European patients than that of their US counterparts (7.37% vs. 9.65%; *p*-value = 0.016). The estimated hazard ratio (HR) was 1.29 (95% CI 1.05–1.59).

**Conclusion:**

This large-scale transatlantic comparative analysis shows that there are some significant differences in patient profiles between large cohorts of European and US patients. These differences were adjusted by using PSM for the mortality analysis. No significant difference in mortality was detected between groups through 2 years, but survival was significantly better in the European DuraGraft Registry patients at 3 years post-CABG.

## 1 Introduction

The European and US cardiovascular surgery societies continuously exchange knowledge and best practices to optimize patient standard of care in coronary artery bypass grafting (CABG), which is also reflected in the similarity of current guidelines and their overall coherence (1, 2). However, there is limited evidence of whether such efforts result in similar patient outcomes post-CABG.

Therefore, the main aims of this study were to (1) compare patient profiles and (2) assess the mid-term survival at 3 years post-CABG between European and US patient cohorts, who underwent isolated CABG surgery. Additionally, since all free grafts from the European patients were stored in and flushed with DuraGraft, an endothelial damage inhibitor (EDI), whereas the grafts from the US patients were stored in and flushed with standard-of-care solutions (e.g., saline and heparinized blood), it was important to also understand the potential impact of DuraGraft use on patient outcomes. DuraGraft is an ionically and pH-balanced physiological salt solution and generator of nitric oxide protecting the structure and function of the vascular endothelium and mitigating ischemic and reperfusion damage during graft storage with recent studies suggesting a protective effect (3, 4).

## 2 Methodology

### 2.1 Patients

### 2.2 EU DuraGraft Registry

The EU DuraGraft Registry (NCT02922088) is a registry of patients who have undergone CABG and whose free venous and arterial grafts have been treated with DuraGraft (5–7). Details of the registry design have been published previously (8).

#### 2.2.1 Eligibility criteria

##### 2.2.1.1 Inclusion criteria

• Patient is undergoing isolated CABG procedure or CABG plus aortic or mitral valve surgery with at least one saphenous vein or radial artery graft
• Patient is ≥18 years of age
• Patient (or a legally authorized representative) is willing and able to provide consent
• DuraGraft is being used for the CABG procedure

##### 2.2.1.2 Exclusion criteria

• Participation in a device study or receiving active drug product in an investigational study within 1 month (30 days) prior to enrollment.

A total of 2,964 patients were enrolled between December 2016 and August 2019 at 45 sites in eight European countries, namely, Austria, Germany, Ireland, Italy, Spain, Switzerland, Turkey, and the United Kingdom. Of these, a total of 2,522 patients underwent isolated CABG.

### 2.3 STS Registry

The STS database is the world's premier and largest clinical outcomes registry for adult cardiac surgery (9). It is US-based with approximately 150,000 annual isolated CABG surgeries and thus includes 600,000 patients operated on between 2016 and 2019. More than 95% of US hospitals performing CABG surgery enter data, with 98% of CABG surgeries in the USA captured. Data quality is ensured by built-in adjudication processes, and 10% of sites are randomly audited every year by independent external auditors. Concordance rates between the medical record and submitted data elements consistently exceed 95%.

The case report form (CRF) for the STS database is extensive with a large number of recorded variables matching those in the EU DuraGraft Registry. These included patient demographics, cardiac risk factors, previous cardiac interventions, preoperative cardiac status and medications, hemodynamics, imaging and angiographic studies, operative details on technique (surgical approach, on- vs. off-pump use, use of cardioplegia), grafts used [left internal mammary artery (LIMA), right internal mammary artery (RIMA), radial artery], number of distal anastomoses, concomitant procedures, assist devices, postoperative course details, hospital discharge information, and outcomes to 30 days. In addition, the patients in the STS Registry have been matched to the US National Death Index up to those who died in 2020, enabling accurate assessment of mortality beyond the 30 days of mortality present in the STS database. Since no long-term data on the occurrence of myocardial infarction or the need for repeat revascularization are available from the STS database, our analysis at this time was limited to overall mortality.

### 2.4 Approach

#### 2.4.1 Patient profile comparison

Patient profiles including demographics, risk factors, and procedural characteristics from the EU DuraGraft Registry were compared to data from the patient profiles from the STS Registry Adult Cardiac Surgery Database (ACSD).

#### 2.4.2 Endpoint

The primary endpoint was mortality through 3 years of follow-up. The STS database contains data through 30 days after CABG surgery. STS's data were merged with the National Death Index, a database maintained by the National Center for Health Statistics (NCHS), which captures all death records for the USA and US territories, allowing for mortality to be assessed for most STS patients beyond 30 days.

We used propensity matching to balance demographic and procedural characteristics in patients in the EU DuraGraft Registry compared to a matched group of patients who received standard-of-care surgical treatment in the STS ACSD Database.

The variables included in the propensity score were prespecified and chosen to reflect mortality risk in the operative, perioperative, and follow-up periods. The time period of 3 years extends beyond the perioperative period, a time period at which the mortality is largely due to periprocedural and early postoperative events.

The matching algorithm used a greedy algorithm with a caliper of 0.2× SD for logit propensity score with matched STS subjects not replaced. The closest match was selected, and if multiple subjects were available for match, the subjects were chosen randomly based on a uniform random number with a prespecified seed (subject ID).

### 2.5 Analysis set

For this analysis, we considered all patients (*N* = 2,522) from the EU DuraGraft Registry who underwent isolated CABG surgery and adult patients (*N* = 294,725) from the STS Adult Cardiac Surgery Database (ACSD) who underwent isolated CABG surgery between 2016 and 2019 at US sites and who met the inclusion/exclusion criteria of the EU DuraGraft Registry, with matching National Death Index data and with non-missing data for the pre-selected variables for the propensity score variables.

#### 2.5.1 Multistep approach

To avoid potential bias, we adopted a multistep approach for the analysis of data as outlined below:

1. The validated data for the chosen propensity score variables for subjects from the European DuraGraft Registry, without any outcomes data, was transferred to the STS analysis team.
2. Using solely the prespecified set of covariates, two propensity scores were estimated based on multivariable logistic regression models:
  • Primary propensity score model: estimated based on 35 prespecified variables (listed in Supplementary Material), including demographics, cardiac and preoperative surgical risk factors, coronary anatomy, and surgical/procedural key characteristics (e.g., grafting strategy and conduit selection) to serve as the primary analysis.
  • Secondary propensity score model: estimated based on 25 prespecified variables (listed in Supplementary Material), which included the set of variables included in the primary propensity score model without intraoperative factors of patient risk and surgical characteristics.
3. STS analysis team then:
  • Estimated the propensity scores based on the two models.
  • Checked the overlap between the propensity scores of the two cohorts using estimated densities and numerical summaries.
  • Checked with the European DuraGraft Registry on the propensity score appropriateness.
  • Performed the match. The matching algorithm used a greedy algorithm with a caliper of 0.2× SD for logit propensity score with matched STS subjects not replaced. The closest match was selected, and if multiple subjects were available for match, the subjects were chosen randomly based on a uniform random number with a prespecified seed (subject ID).
  • For all the variables included in the propensity score plus additional variables provided by the European DuraGraft Registry, the quality of the match was checked using the following:
    ◦ Table with standardized differences
    ◦ Side-by-side boxplots
    ◦ Overlayed density plots
    ◦ Quantile–quantile plots
4. To avoid potential bias, a blinding procedure was protocolized whereby the STS team did not have access to any outcome data from the EU DuraGraft Registry until after the propensity match cohorts were finalized.
5. After matching was performed, the DuraGraft outcome data were transferred to the STS analysis team.
6. Outcome analysis was performed using matches based on both primary and secondary propensity models.
7. STS performed analyses contrasting STS and DuraGraft Registry mortality data through 3 years. The analyses included Kaplan–Meier (KM) plots and stratified Cox regression, with strata defined by matched pairs. The hazard ratio (HR) comparing the mortality hazard in the DuraGraft with the hazard in the STS Registry was reported along with a 95% confidence interval.

### 2.6 Prespecified propensity score variables

The goal of propensity matching was to balance patient and technical factors predictive of mortality throughout the period of observation, to correct for differences that may be encountered in the USA and Europe. An important set of variables that needed to be balanced were the components of the EuroScore II (ESII). ESII, comprised of 18 patient variables, is considered to be the best predictor of perioperative and early mortality. ESII variables relevant for shorter-term mortality were supplemented with appropriate predictors for longer-term mortality.

The set of variables for the primary propensity score model included 35 characteristics that are most strongly associated with mortality across the time periods (including long-term post-CABG) and were consistently observed to have the highest degree of impact in the studies. To further allow for the selection of a cohort matched for standard of care and surgical technique between the European and US populations, additional relevant variables were added consisting of factors of preoperative cardiac risk, coronary anatomy, and surgical technique (Supplementary Table S1).

The set of variables for the secondary propensity score model included 25 of the 35 variables from the primary propensity score model, minus characteristics of preoperative cardiac risk factors, coronary anatomy, and aspects of surgical technique. This model serves as a sensitivity analysis to estimate whether standard of care for the treatment of patients with advanced coronary artery disease and surgical techniques differ in patients otherwise balanced for surgical risk factors and whether these differences could affect mortality outcomes (Supplementary Table S1).

Love plots were generated to demonstrate quality of matching (Supplementary Figure S1).

### 2.7 Analysis set

• EU DuraGraft Registry: all patients who underwent isolated CABG surgery (*N* = 2,522)
• STS Adult Cardiac Surgery Database (ACSD): adult patients who underwent isolated CABG surgery between 2016 and 2019 at US sites (*N* = 294,725)

### 2.8 Analysis software

SAS software, version 9.4 (SAS Institute), was used for matching and R [R Core Team (2023). R: A Language and Environment for Statistical Computing. R Foundation for Statistical Computing, Vienna, Austria] for statistical analyses and graphs. Statistical significance was considered for *p* < 0.05.

## 3 Results

### 3.1 Patient profile comparison

The detailed patient profile comparison is summarized in Table 1.

**Table 1:** Patient profiles before propensity score matching.

Variable	Unmatched cohorts			
	STS	DuraGraft	*p*	SMD (%)
No. of patients (*N*)	294,725	2,522		
Age	65.6 ± 9.9	67.3 ± 9.2	<0.001	18.1
Male sex	76.1%	82.5%	<0.001	15.7
Black race	7.5%	0.1%	<0.001	39.2
BMI < 20 kg/m^2^	1.7%	0.7%	<0.001	8.9
Previous/current smoker	61.4%	62.1%	0.467	1.5
Diabetes—insulin	18.6%	14.5%	<0.001	11.1
Diabetes—no insulin	31.6%	28.8%	0.002	6.2
CRF (Cr > 2.0 mg/dl)	2.0%	2.2%	0.474	1.5
Dialysis	3.1%	1.1%	<0.001	13.4
PVD	22.0%	16.1%	<0.001	15.1
PH	19.3%	8.2%	<0.001	32.9
History of PD	15.9%	13.9%	0.007	5.6
History of CVA	11.2%	7.9%	<0.001	11.3
MI ≤ 24 h	3.2%	1.4%	<0.001	12.0
MI > 24 h	50.9%	41.4%	<0.001	19.1
Unstable angina	34.9%	8.9%	<0.001	66.2
Heart failure	24.6%	14.2%	<0.001	26.4
Cardiogenic shock	1.3%	0.9%	0.077	4.0
Preop atrial fibrillation	9.7%	7.8%	0.002	6.5
Reoperation	1.6%	1.3%	0.193	2.9
Left main stem disease	26.1%	40.9%	<0.001	31.8
Three-vessel disease	79.6%	81.2%	0.055	3.9
LVEF (<30%)	6.1%	3.1%	<0.001	14.7
Status urgent	60.0%	24.1%	<0.001	78.0
Status emergent	3.7%	1.3%	<0.001	14.9
Previous CABG^a^	1.3%	0.3%	<0.001	11.3
Previous PCI^a^	30.0%	24.6%	<0.001	12.1
Intraoperative factors				
On-pump status^a^	88.8%	82.9%	<0.001	17.0
No distal anastomoses^a^	3.41 ± 1.0	3.0 ± 0.9	<0.001	43.2
Use of LIMA graft^a^	97.3%	90.8%	<0.001	27.5
No. of arterial grafts^a^	1.1 ± 0.5	1.3 ± .0.7	<0.001	28.5
No. of venous grafts^a^	2.2 ± 1.0	1.5 ± 0.9	<0.001	76.2
All-arterial grafts^a^	2.0%	10.4%	<0.001	35.6
All venous grafts^a^	2.7%	5.3%	<0.001	13.1
Endoscopic harvest^a^	89.9%	13.8%	<0.001	235.1

^a^ Parameters only included in the primary model.

CABG, coronary artery bypass grafting; CRF, case report form; LIMA, left internal mammary artery; CVA, cerebrovascular accident; LVEF, left ventricular ejection fraction; PCI, percutaneous coronary intervention; PD, pulmonary disease; PH, pulmonary hypertension; PVD, peripheral vascular disease; SMD, standard mean difference; STS, Society of Thoracic Surgeons.

In summary, the analysis showed that the US and European cohorts show some significant differences in demographics and pre- and intraoperative variables. In brief, while US patients received more distal anastomoses with more saphenous vein grafts (SVGs) that were primarily harvested endoscopically, EU patients presented with more left main disease (LMD) disease, underwent more off-pump CABG, and received more arterial grafts together with more all-arterial grafting procedures.

After robust propensity score matching (PSM), the observed differences were well balanced to allow for the comparative mortality analysis (Table 2).

**Table 2:** Patient profiles after propensity score matching with the secondary model (25 parameters matched) and the primary model (35 parameters matched).

Variable	Matched cohorts—secondary model				Matched cohorts—primary model			
	STS	DuraGraft	*P*	SMD (%)	STS	DuraGraft	*p*	SMD (%)
No. of patients (*N*)	2,522	2,522			2,400	2,400		
Age	67.0 ± 9.3	67.4 ± 9.2	0.251	3.2	67.3 ± 9.2	67.3 ± 9.8	0.989	−0.0
Male sex	82.5%	82.5%	1.00	<0.1	82.1%	82.7%	0.596	−1.5
Black race	0.25%	0.1%	0.317	−2.8	0.1%	0.1%	0.655	1.3
BMI < 20 kg/m^2^	0.7%	0.7%	1.00	<0.1	0.7%	0.6%	0.589	1.6
Previous/current smoker	63.4%	62.1%	0.351	−2.6	61.9%	63.4%	0.283	−3.1
Diabetes—insulin	13.3%	14.5%	0.222	3.4	14.6%	13.8%	0.408	2.4
Diabetes—no insulin	29.5%	28.8%	0.598	−1.5	29.1%	28.6%	0.702	1.1
CRF (Cr > 2.0 mg/dl)	2.2%	2.2%	1.00	<0.1	2.2%	2.3%	0.771	−0.8
Dialysis	0.9%	1.1%	0.325	2.8	1.2%	1.3%	0.795	−0.8
PVD	15.3%	16.1%	0.486	2.0	16.3%	16.9%	0.587	−1.6
PH	8.0%	8.2%	0.877	0.4	8.6%	8.3%	0.677	1.2
History of PD	14.0%	13.9%	0.871	−0.5	14.0%	13.9%	0.901	0.4
History of CVA	6.3%	7.9%	0.037	5.9	8.0%	8.4%	0.598	−1.5
MI ≤ 24 h	1.3%	1.4%	0.712	1.0	1.4%	0.9%	0.107	4.7
MI > 24 h	42.5%	41.4%	0.441	−2.2	41.9%	41.0%	0.538	1.8
Unstable angina	9.4%	8.9%	0.558	−1.6	9.4%	9.7%	0.694	−1.1
Heart failure	13.7%	14.2%	0.597	1.5	14.6%	14.0%	0.536	1.8
Cardiogenic shock	0.7%	0.9%	0.341	2.7	1.0%	0.8%	0.535	1.8
Preop atrial fibrillation	6.6%	7.8%	0.103	4.6	8.0%	9.8%	0.029	−6.3
Reoperation	1.2%	1.3%	0.798	0.7	1.2%	1.2%	1.000	0.0
Left main stem disease	41.0%	40.9%	0.954	−0.2	39.2%	39.7%	0.723	−1.0
Three-vessel disease	81.9%	81.2%	0.537	−1.7	80.7%	81.1%	0.713	−1.1
LVEF (<30%)	2.4%	3.1%	0.167	3.90	3.2%	3.3%	0.870	−0.5
Status urgent	23.9%	24.1%	0.895	0.4	25.1%	25.5%	0.791	−0.8
Status emergent	1.4%	1.3%	0.904	−0.3	1.4%	1.1%	0.367	2.6
Previous CABG^a^	1.2%	0.3%	<0.001	−10.4	0.3%	0.3%	0.796	0.7
Previous PCI^a^	30.5%	24.6%	<0.001	−13.2	24.9%	24.5%	0.763	0.9
Intraoperative factors								
On-pump status^a^	89.1%	82.9%	<0.001	−17.8	83.8%	84.9%	0.284	−3.1
No distal anastomoses^a^	3.4 ± 1.0	3.0 ± 0.9	<0.001	−44.5	3.0 ± 0.9	3.0 ± 1.1	0.507	−1.9
Use of LIMA graft^a^	97.9%	90.8%	<0.001	−31.1	92.6%	92.3%	0.623	1.4
No. of arterial grafts^a^	1.1 ± 0.5	1.3 ± 0.7	<0.001	22.3	1.3 ± 0.7	1.3 ± 0.8	0.805	−0.7
No. of venous grafts^a^	2.2 ± 1.0	1.5 ± 0.9	<0.001	−72.8	1.5 ± 0.9	1.6 ± 0.9	0.809	−0.7
All-arterial grafts^a^	2.8%	10.4%	<0.001	31.2	10.6%	10.8%	0.852	−0.5
All venous grafts^a^	2.1%	5.3%	<0.001	16.8	4.9%	5.1%	0.791	−0.8
Endoscopic harvest^a^	91.0%	13.8%	<0.001	−244.2	14.5%	14.6%	0.870	−0.5

^a^ Parameters only included in the primary model.

CABG, coronary artery bypass grafting; CRF, case report form; LIMA, left internal mammary artery; CVA, cerebrovascular accident; LVEF, left ventricular ejection fraction; PCI, percutaneous coronary intervention; PD, pulmonary disease; PH, pulmonary hypertension; PVD, peripheral vascular disease; SMD, standard mean difference; STS, Society of Thoracic Surgeons.

### 3.2 Primary outcome

**Figure 1:**
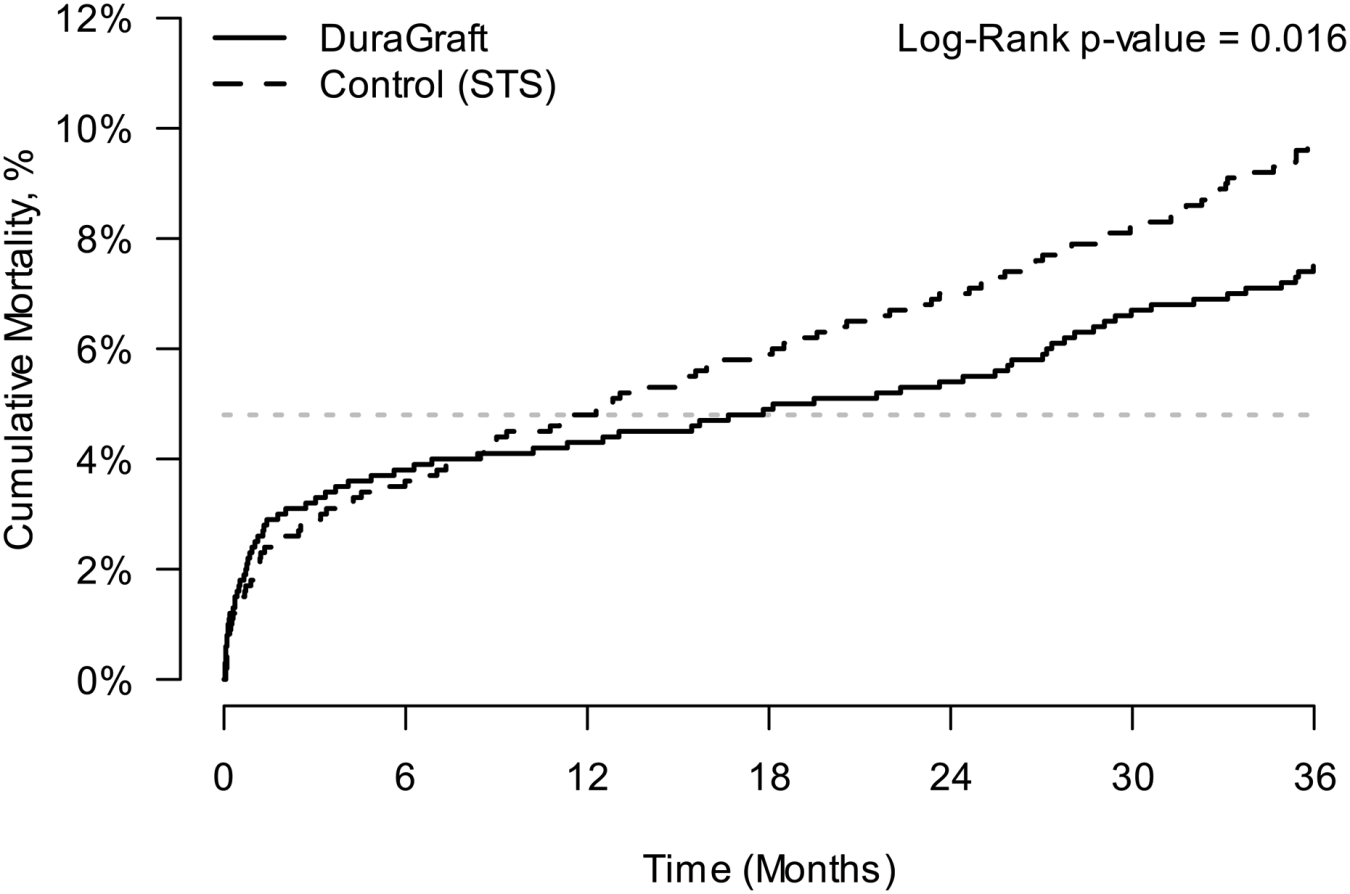
Kaplan–Meier estimate of cumulative incidence of all-cause mortality in the two matched cohorts; matching done based on the primary propensity score model.

Mortality outcomes were measured through 3 years. The cumulative incidence of mortality through 3 years was estimated in the two matched groups (2,400 patients in each group) using Kaplan–Meier (KM) curves (Figure 1). At 30 days, 12 months, and 24 months, the mortality estimate in patients from the European DuraGraft Registry was 2.38% (95% CI 1.84%–3.07%), 4.32% (95% CI 3.58%–5.22%), and 5.38% (95% CI 4.58%–6.42%) and that in the STS Registry patients was 1.96% (95% CI 1.47%–2.60%), 4.79% (95% CI 4.01%–5.72%), and 6.96% (95% CI 5.98%–8.06%), respectively, without showing any statistically significant difference.

At 36 months, the mortality estimate in European DuraGraft Registry patients was significantly lower when compared to STS patients [7.37% (95% CI 6.36%–8.53%) vs. 9.65% (95% CI 8.37%–11.10%); log-rank *p*-value = 0.016].

Using a stratified Cox regression model, a 29% increase in hazards for mortality was estimated [HR = 1.29 (95% CI 1.05–1.59)].

#### 3.2.1 Sensitivity analysis results: safety assessment based on matching using the secondary propensity score model (25 parameters)

**Figure 2:**
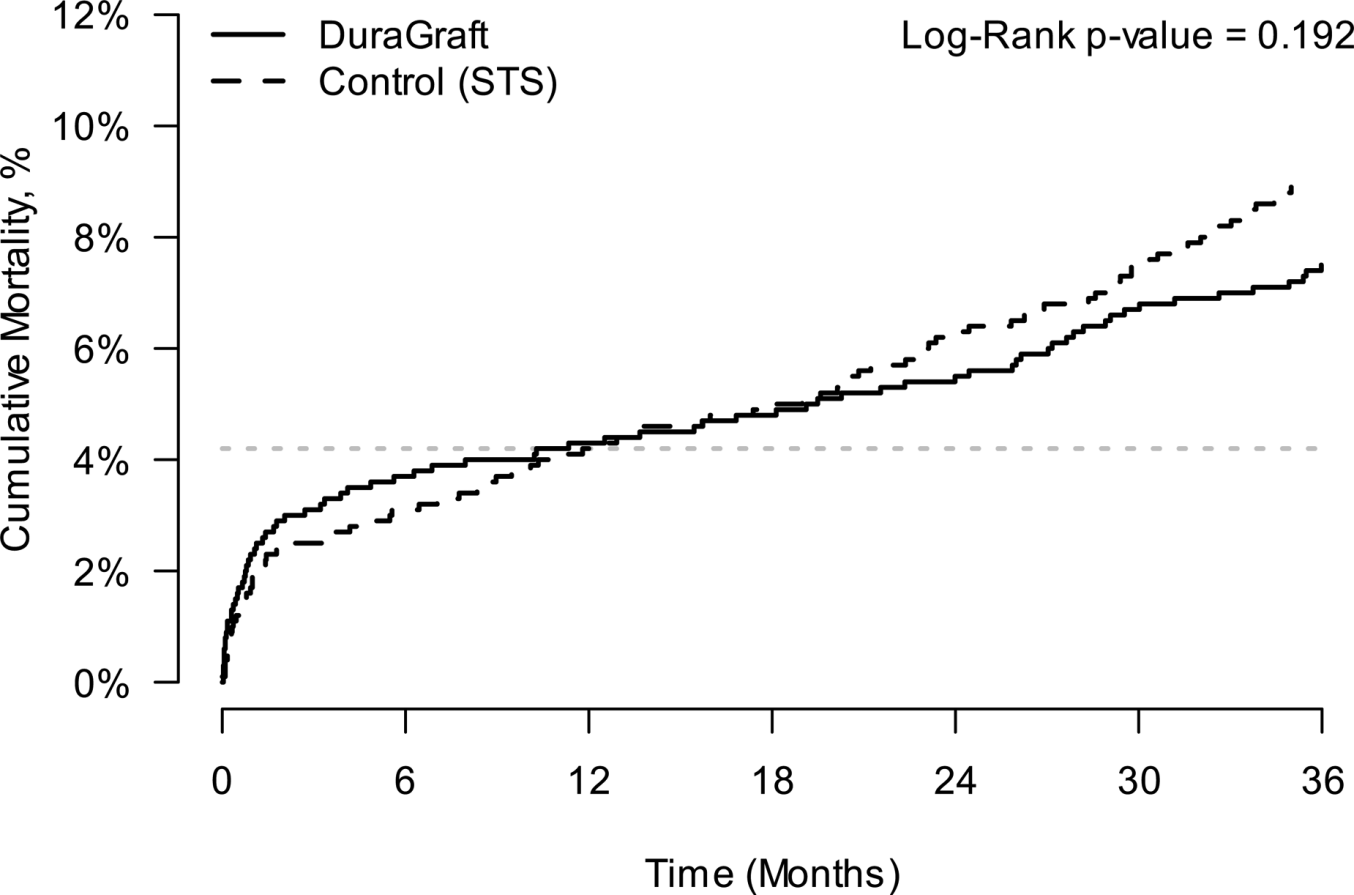
Kaplan–Meier estimates of cumulative incidence of all-cause mortality in matched cohorts; matching based on the secondary propensity score model.

A sensitivity analysis was conducted using matching based on propensity scores estimated in the secondary propensity score model (Table 2, 25 parameters matched) to demonstrate the robustness of the safety analysis results obtained based on the matching using the primary propensity score model. The secondary propensity score model used 25 variables instead of 35, with fewer cardiac risk factors and a lack of balance for surgical technique. A total of 2,522 patients from each cohort were matched. The cumulative incidence of mortality through 3 years was estimated in the two matched groups using Kaplan–Meier (KM) curves (Figure 2).

At 36 months, the mortality estimate in European patients was 7.39% (95% CI 6.40%–8.52%) compared to 8.89% (95% CI 7.65%–10.31%) in the STS Registry patients, log-rank *p*-value = 0.192, a non-statistically significant reduction.

Based on a stratified Cox regression model, comparing the European patients with the US patients, the estimated hazard ratio (HR) was 1.15 (95% CI 0.93–1.42).

Taken together, for both PSM models, while there were numerical differences in survival, there was no significant difference between the European and US cohorts throughout 2 years post-CABG. However, a significant difference in mortality was seen at 3 years in favor of the European DuraGraft Registry patients.

## 4 Discussion

In this large-scale transatlantic comparative analysis, we found that there are some significant differences between European and US patients with regard to demographics and pre- and intraoperative variables. Our results show that US patients received more distal anastomoses with more SVGs that were primarily harvested endoscopically. Instead, European patients suffered more frequently from left main disease (LMD), had more often off-pump CABG, and appeared to receive more multi- or all-arterial grafting procedures.

When accounting for these differences by applying a robust PSM model, our comparative analysis showed non-significant differences in mortality rates throughout 2 years after CABG suggesting a transatlantic coherence in quality and outcome. This equivalence was evident irrespective of the inclusion of surgical variables in the PSM (primary model), thus demonstrating the robustness of the findings. While these results can certainly be substantially attributed to the overall coherence between US and European guidelines, they also emphasize the continuous need for the exchange of data and knowledge between Europe and the USA to achieve and maintain consensus on best practices in CABG.

The observed outcome difference between the primary and secondary model (which was used as a sensitivity analysis) may suggest the importance of also including surgical parameters (primary model) in the analysis when assessing for outcomes after CABG. In fact, the secondary analysis only included patient demographics and risk factors but did not match for all factors of surgical technique and underlying patient anatomy, perhaps resulting in a comparison of two cohorts that may have had an unbalanced surgical risk.

European patients fared better at 3 years post-CABG. Interestingly, it is to be recognized that in comparison to their US counterparts, all free grafts of European patients were flushed and stored in with DuraGraft, an EDI, before distal anastomosis. However, while the systematic use of DuraGraft, an EDI, appears to have an impact on better survival in European patients, it will be interesting and important to assess this in future studies (10).

Our findings are consistent with a recently published article by Lopez-Menendez et al. (11) who conducted a small observational, prospective, longitudinal, single-center study in patients undergoing isolated CABG. The authors showed that the use of DuraGraft was associated with a significantly decreased incidence of major adverse cardiac events (MACE) at 3 years after CABG including a significantly better overall survival. This protective effect was particularly pronounced in diabetic patients and those who received multiple SVGs.

Recently, researchers increasingly dedicated efforts to understanding the underlying mechanisms and effects of the systematic use of EDI for conduits used in CABG procedures. As such, Tekin et al. (12) showed that saphenous vein grafts stored in DuraGraft had a lower oxidative level, higher antioxidant level, and a lower oxidative stress index in comparison to saphenous vein grafts stored in saline or heparinized blood. Aschacher et al. (13) concluded that EDI treatment with DuraGraft preserves the functionality and integrity of endothelial and intimal cells with the potential to reduce the occurrence of graft disease and failure in grafts in patients undergoing CABG. In a recent study, researchers demonstrated the protective effects of DuraGraft with regard to the connectivity and function of the vein graft endothelium by preserving focal adhesions in venous endothelial cells during short-term storage after graft harvesting, thus maintaining most of the endothelium in venous CABG surgery conduits (7).

While arterial grafting is widely believed to be associated with improved outcomes and thus endorsed by the European Association for Cardio-Thoracic Surgery (EACTS) and the Society of Thoracic Surgeons (STS) in their recent expert systematic review (14), its use in the USA still appears rather low with <7% in routine CABG practice according to the STS Adult Cardiac Surgical Database (ACSD). In line with this, in our present analysis, the use of multi- or all-arterial grafting procedures was lower in US patients when compared to European patients. However, such differences were balanced in our primary analyses, and therefore such differences in arterial grafting are not believed to explain the difference in mortality.

This study has several limitations: since no long-term data on the occurrence of myocardial infarction and the need for repeat revascularization were not available from the STS database, our analysis was limited to overall mortality. Moreover, while every free graft in the European cohort was systematically stored in and flushed with DuraGraft, detailed information on the type and distribution of the utilized standard-of-care solutions (e.g., saline, heparinized saline or blood, and buffered saline) in the US cohort was not available. Finally, a comparison for postoperative antiplatelet management that is known to have an influence on outcomes was not possible as such data were not available for the US and European cohorts.

## 5 Conclusions

This large-scale transatlantic comparative analysis shows that there are some significant differences in patient profiles between large cohorts of European and US patients. These differences were balanced using robust PSM. While numerical differences in mortality were observed no significant difference in mortality was detected throughout 2 years. Survival, however, was significantly better in European DuraGraft Registry patients at 3 years post-CABG. While it appears that the use of DuraGraft had a positive impact on the outcomes observed in the European cohort, the extent to which and mechanisms by which the systematic use of an EDI may improve survival remains to be elucidated in further studies. Further systematic studies are warranted to follow up on these questions and also to inquire about the effects of EDI on the occurrence of myocardial infarction and the need for revascularization following CABG.

## Registration

The European Multicenter Registry to Assess Outcomes in CABG Patients is registered at ClinicalTrials.gov (NCT02922088) and is accessible at https://clinicaltrials.gov/ct2/show/NCT02922088.

## Data Availability

The original contributions presented in the study are included in the article/Supplementary Material, further inquiries can be directed to the corresponding author.
