# Supplementary material for: Transatlantic analysis of patient profiles and mid-term survival after isolated coronary artery bypass grafting: a head-to-head comparison between the European DuraGraft Registry and the US STS Registry
Source: Front Cardiovasc Med. 2024 Sep 12;11:1366460. doi: 10.3389/fcvm.2024.1366460 (PMC11428045; doi:10.3389/fcvm.2024.1366460)

SUPPLEMENTARY MATERIALS

Transatlantic analysis of patient profiles and mid-term overall survival after isolated coronary artery bypass grafting: a head-to-head comparison between the European DuraGraft Registry and the US STS Registry

Etem Caliskan[1], Martin Misfeld, MD, PhD[2-6], Sigrid Sandner[7,8], MD, Andreas Böning, MD[9], Jose Aramendi, MD[10], Sacha P Salzberg, MD[11], Yeong-Hoon Choi, MD[12], Louis P Perrault, MD, PhD[13], Ilker Tekin, MD[14,15], Gregorio P. Cuerpo, MD[16], Jose Lopez-Menendez, MD, PhD[17], Luca P. Weltert, MD[18], Johannes Böhm, MD, PhD[19], Markus Krane, MD, PhD[20], José M. González-Santos, MD, PhD[21], Juan-Carlos Tellez, MD[22], Tomas Holubec, MD, PhD[23], Enrico Ferrari, MD, PhD[24], Gheorghe Doros, PhD[25], Maximilian Y Emmert, MD, PhD[7,8] on behalf of the Registry Investigators

REGISTRY ADVISORY COMMITTEE

Maximilian Y Emmert (Chair)	Department of Cardiovascular Surgery, Charité Universitätsmedizin Berlin, Berlin, Germany
	Department of Cardiothoracic and Vascular Surgery, German Heart Center Berlin, Berlin, Germany
Jose I. Aramendi	Division of Cardiac Surgery, Hospital de Cruces, Barakaldo, Spain
Andreas Böning	Universitätsklinikum Gießen und Marburg GmbH, Gießen, Germany
Etem Caliskan	Department of Cardiovascular Surgery, Charité Universitätsmedizin Berlin, Berlin, Germany
	Department of Cardiothoracic and Vascular Surgery, German Heart Center Berlin, Berlin, Germany
Yeong-Hoon Choi	Kerckhoff Heart Center Bad Nauheim, Campus Kerckhoff Justus-Liebig University Giessen
Martin Misfeld	University Department of Cardiac Surgery, Leipzig Heart Center, Leipzig, Germany
	Department of Cardiothoracic Surgery, Royal Prince Alfred Hospital, Sydney, Australia
	Institute of Academic Surgery at RPA, Sydney, Australia
	The Baird Institute of Applied Heart and Lung Surgical Research, Sydney, Australia
	Medical School, University of Sydney, Australia
Sacha Salzberg	Swiss Heart Clinic, Zurich, Switzerland
Sigrid Sandner	Department of Cardiac Surgery, Vienna General Hospital, Medical University of Vienna, Vienna, Austria
Louis P. Perrault	Montreal Heart Institute, Montreal, Canada

CLINICAL EVENTS COMMITTEE

Jacques J Koolen	Catharina Hospital, Eindhoven, the Netherlands
Pascal Vranckx	Department of Cardiology and Critical Care Medicine, Hartcentrum Hasselt, Jessa Ziekenhuis, Belgium<br>Faculty of Medicine and Life Sciences, Hasselt University, Belgium

LIST OF INVESTIGATORS AND COLLABORATORS

Country/centre	Investigators	Nº of patients
**AUSTRIA**		**269**
Medical University Vienna	Sigrid Sandner<br>Daniel Zimpfer	225
Medical university of Innsbruck	Ulvi Cenk Oezpeker<br>Michael Grimm	10
Departement Hospital Clinic Floridsdorf/Vienna Heart Center	Bernhard Winkler<br>Martin Grabenwöger	14
Klinikum Klagenfurt am Wörthersee	Michaele Andrä	20
**GERMANY**		**985**
University Hospital Schleswig-Holstein, Campus Lübeck	Anas Aboud<br>Stephan Ensminger	20
University Department of Cardiac Surgery, Leipzig Heart Center, Leipzig, Germany	Martin Misfeld<br>Michael A. Borger	248
Universitätsklinikum Gießen und Marburg GmbH	Andreas Böning<br>Bernd Niemann	160
University Hospital Frankfurt	Tomas Holubec<br>Arnaud Van Linden	81
West-German Heart and Vascular Center, University Hospital Essen, University Duisburg-Essen	Matthias Thielmann<br>Daniel Wendt	55
Universitätsklinikum Schleswig-Holstein Campus Kiel	Assad Haneya	7

	Katharina Huenges	
German Heart Center Munich	Johannes Böhm<br>Markus Krane	115
Charité - Universitätsmedizin Berlin, corporate member of Freie Universität Berlin and Humboldt-Universität zu Berlin	Etem Caliskan<br>Herko Grubitzsch	182
Universitätsklinikum Wuppertal	Farhad Bakthiary	7
Deutsches Herzzentrum Berlin	Jörg Kempfert<br>Adam J. Penkalla	32
University Medical Center Göttingen	Bernhard C. Danner<br>Fawad A. Jebran	29
RWTH Aachen University	Carina Benstoem<br>Andreas Goetzenich<br>Christian Stoppe	12
University of Cologne	Elmar W. Kuhn<br>Yeong-Hoon Choi<br>Oliver J. Liakopoulos	5
Heart Center Dresden	Stefan Brose<br>Klaus Matschke	32
**IRELAND**		**31**
University Hospital Galway	Dave Veerasingam	27
Cork University Hospital	Kishore Doddakula	4
**ITALY**		**176**
European Hospital, Rome	Luca P. Weltert<br>Lorenzo Guerrieri Wolf	118
Magna Graecia University of Catanzaro	Giuseppe Filiberto Serraino<br>Pasquale Mastroroberto	48
ULSS 8 Berica, Vicenza	Nicola Lamascese<br>Massimo Sella	10
**SPAIN**		**1081**
Hospital Universitario Ramon y Cajal, Madrid	Jose Lopez-Menendez	125

	Edmundo R. Fajardo-Rodriguez	
Cruces University Hospital, Barakaldo Bizkaia	Jose I. Aramendi<br>Alejandro Crespo	50
Hospital Universitario Santiago de Compostela	Angel L Fernandez Gonález	84
Hospital General Universitario Gregorio Marañón, Madrid	Gregorio P. Cuerpo<br>Alvaro Pedraz	125
Hospital Universitario de Salamanca	José M. González-Santos<br>Elena Arnáiz-García	100
Hospital Universitario Reina Sofia, Córdoba	Ignacio Muñoz Carvajal	56
Hospital Universitario Puerta del Mar, Cadiz	Adrian J. Fontaine	63
Complejo Hospitalario Universitario de Badajoz	José Ramón González Rodríguez	192
	José Antonio Corrales Mera	
Complejo hospitalario Ruber Juan Bravo, Madrid	Paloma Martinez	25
Hospital Universitario La Paz, Madrid	Jose Antonio Blazquez	2
Hospital Universitario Virgen Macarena, Seville	Juan-Carlos Tellez<br>Bella Ramirez	94
Virgen del Rocio University Hospital, Seville	Alejandro Adsuar-Gomez<br>Jose M. Borrego-Dominguez	61
Hospital Universitario Germans Trias y Pujol, Barcelona	Christian Muñoz-Guijosa<br>Sara Badía-Gamarra	40
Complejo Hospitalario de Navarra/Navarra Biomed, Pamplona	Rafael Sádaba<br>Alicia Gainza	13
Clinic Hospital, University of Barcelona	Manuel Castellá	28
Hospital Universitario de León	Gregorio Laguna<br>Javier A. Gualis	23
**SWITZERLAND**		**133**
Cardiocentro Ticino Institute, Lugano	Enrico Ferrari<br>Stefanos Demertzis	80

Swiss Heart Clinic AG, Zurich	Sacha Salzberg<br>Jürg Grünenfelder	4
University Hospital of Zurich	Robert Bauernschmitt	49
**TURKEY**		**160**
Manavgat Government Hospital	Ilker Tekin	160
**UNITED KINGDOM**		**126**
Blackpool Teaching Hospitals	Amal K. Bose	60
Golden Jubilee National Hospital, Glasgow	Nawwar Al-Attar<br>George Gradinariu	66

Suppl. Table 1: Variables Included in Primary and Secondary Propensity Score Models

	Primary Model (35 variables)	Secondary Model (25 variables)
**Demographics**		
Age*	X	X
Male sex*	X	X
Black race	X	X
**Cardiac Risk Factors**		
BMI < 20 kg/m^2	X	X
Previous or Current Smoker	X	X
Diabetes on Insulin*	X	X
Diabetes Not on Insulin	X	X
CRF (Cr > 2.0 mg/dl)*	X	X
Renal Dialysis*	X	X
Peripheral Vascular Disease*	X	X
Pulmonary Hypertension*	X	X
History of Pulmonary Disease*	X	X
History of CVA	X	X
**Pre-Op Cardiac Status**		
MI ≤ 24h*	X	X
MI > 24 h*	X	X
Unstable Angina*	X	X
Congestive Heart Failure	X	X
Cardiogenic Shock*	X	X
Pre-operative Atrial Fibrillation	X	X
Re-operation*	X	X
Left ventricular function (EF<30%)*	X	X
Status Urgent*	X	X
Status Emergent*	X	X
**Coronary Anatomy**		
Left Main Stem Coronary Artery Stenosis ≥ 50%	X	X
Three (3) vessel disease	X	X
Previous CABG	X	
Previous PCI	X	
**Surgical Technique**		
Number of distal anastomoses	X	

	**Primary Model (35 variables)**	**Secondary Model (25 variables)**
On Pump Status	X	
Presence of LIMA graft	X	
Number of arterial grafts	X	
Number of venous grafts	X	
All arterial grafts	X	
All venous grafts	X	
Harvesting Technique – Endoscopic	X	

*EuroScore II variables

Suppl. Figures

Suppl. Figure 1: Loveplot showing quality of matching

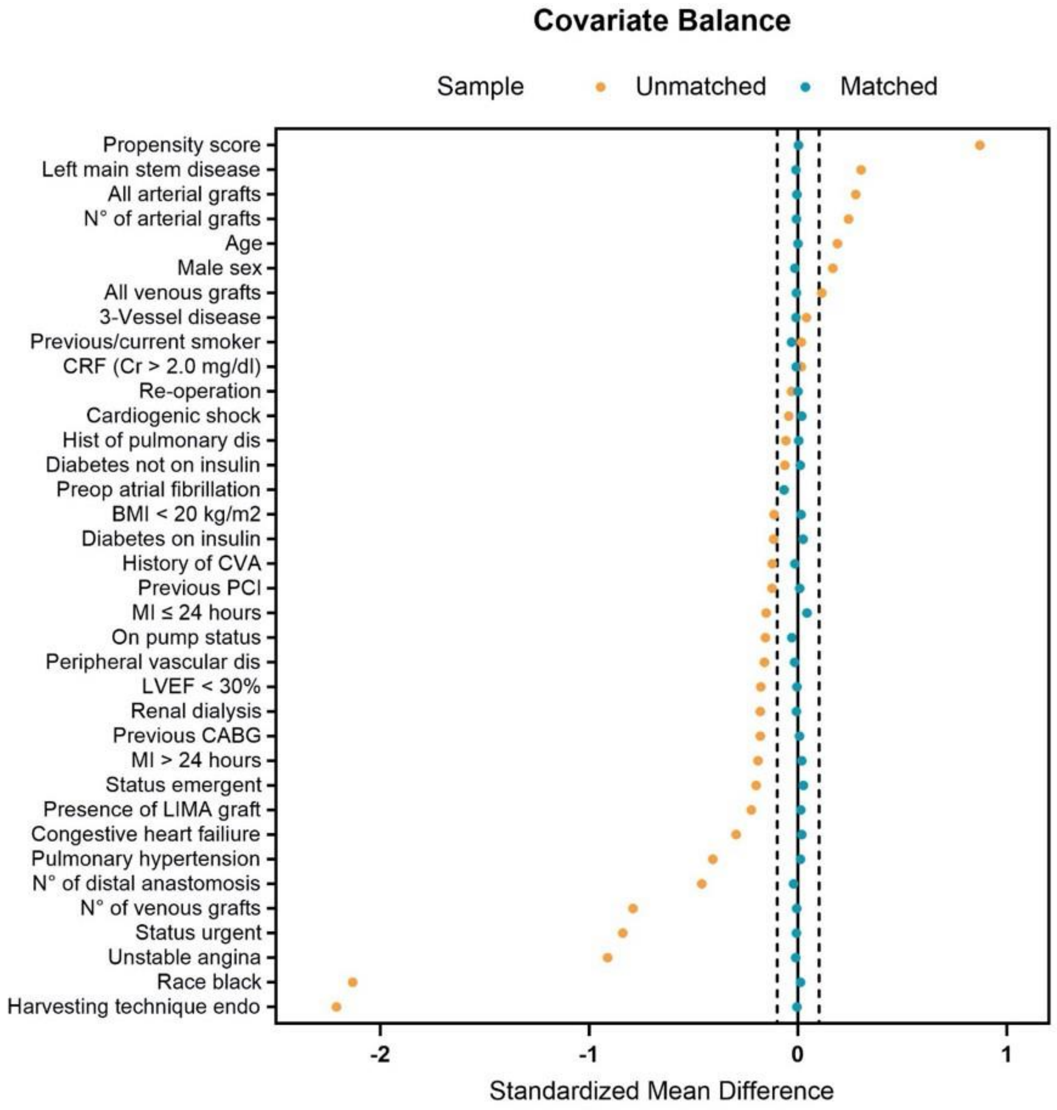

Supplement: Supplementary file 1 [file Datasheet1.pdf]
